# Effectiveness and Cost-Effectiveness of Blended Cognitive Behavioral Therapy in Clinically Depressed Adolescents: Protocol for a Pragmatic Quasi-Experimental Controlled Trial

**DOI:** 10.2196/13434

**Published:** 2019-10-07

**Authors:** Sanne P A Rasing, Yvonne A J Stikkelbroek, Heleen Riper, Maja Dekovic, Maaike H Nauta, Carmen D Dirksen, Daan H M Creemers, Denise H M Bodden

**Affiliations:** 1 Child and Adolescent Studies Utrecht University Utrecht Netherlands; 2 Child and Adolescent Psychiatry GGZ Oost Brabant Boekel Netherlands; 3 The EMGO Institute for Health and Care research Vrije Universiteit Amsterdam Netherlands; 4 Department of Clinical Psychology and Experimental Psychopathology University of Groningen Groningen Netherlands; 5 Academic Centre for Child and Adolescent Psychiatry Groningen Accare Groningen Netherlands; 6 Clinical Epidemiology and Medical Technology Assessment Maastricht University Medical Center Maastricht Netherlands; 7 Developmental Psychopathology Radboud University Nijmegen Netherlands

**Keywords:** depression, major depressive disorder, cognitive behavioral therapy, blended, eHealth, online, adolescents, effectiveness, cost-effectiveness

## Abstract

**Background:**

Cognitive behavioral therapy (CBT) is an effective intervention to treat depressive disorders in youth. However, 50% of adolescents still have depressive symptoms after treatment, and 57% drop out during treatment. Online CBT interventions have proven to be effective in reducing depressive symptoms and seem promising as a treatment for depressed adolescents. However, combining online programs with face-to-face sessions seems necessary to increase their effectiveness and monitor for suicide risk.

**Objective:**

In this study, we examine the effectiveness and cost-effectiveness of a blended CBT treatment protocol, a mixture of online and face-to-face CBT, as a treatment for clinically depressed adolescents.

**Methods:**

A pragmatic quasi-experimental controlled trial will be conducted to study the effectiveness of a blended CBT treatment protocol, in which blended CBT is compared with face-to-face CBT (n=44) and treatment as usual (n=44); the latter two were collected in a previous randomized controlled trial. The same inclusion and exclusion criteria will be used: adolescents aged between 12 and 21 years, with a clinical diagnosis of a depressive disorder, and referred to one of the participating mental health institutions. Assessments will be conducted at the same time points: before the start of the intervention, during the intervention (after 5 and 10 weeks), postintervention, and at 6- and 12-month follow-ups.

**Results:**

The primary outcome is the presence of a depression diagnosis at 12-month follow-up. Several secondary outcomes will be measured, such as depressive symptoms, quality of life, and suicide risk. Costs and effects in both conditions will be compared to analyze cost-effectiveness. Further, moderating (age, gender, alcohol and drug use, parental depression, and other psychopathology) and mediating effects (negative automatic thoughts, cognitive emotion regulation, attributional style) will be analyzed. Also, treatment characteristics will be studied, such as characteristics of the therapists, treatment expectancy, and therapeutic alliance. Dropout rates and treatment characteristics will be measured to study the feasibility of blended CBT.

**Conclusions:**

This study will examine the effectiveness and cost-effectiveness of a blended CBT program in which depressed adolescents are treated in mental health care. Results of blended CBT will be compared with face-to-face CBT and treatment as usual, and implications for implementation will be reviewed.

**Trial Registration:**

Dutch Trial Register (NTR6759); http://www.trialregister.nl/trialreg/admin/rctview.asp?TC=6759

**International Registered Report Identifier (IRRID):**

DERR1-10.2196/13434 RR1-10.2196/12654

## Introduction

Depressive disorders are among the most prevalent mental health disorders in adolescents [1]. They have a substantial effect on well-being, a high burden of disease, and a high risk of recurrence and chronicity [1,2]. At the age of 18 years, more than 15% of adolescents have suffered from a major depressive disorder [3]. Depression shows high comorbidity with other mental health diagnoses and increased social problems, decreased academic performance, increased school dropouts, more substance abuse, and an increased risk of suicide attempts and suicide [1,2,4]. Therefore, it is crucial that depressive disorders are treated effectively at an early age. In this study, we will test the effectiveness and cost-effectiveness of a blended treatment protocol [5] that combines internet-based and face-to-face sessions in an integrated treatment protocol targeting depressive symptoms in clinically depressed adolescents.

Several meta-analyses have shown that cognitive behavioral therapy (CBT) is an effective intervention to treat adolescent depression [6,7]. However, the effect size of CBT was found to be moderate (Cohen’s *d*=0.53) [7], and 50% of the adolescents were not free of depressive symptoms after being treated [8]. A recent Dutch randomized controlled trial (RCT) showed that the D(o)epression protocol (face-to-face individual CBT) was equally effective as treatment as usual (TAU; for example, interpersonal therapy, family therapy, medication, mindfulness training, creative therapy, and running therapy) in reducing depressive symptoms, as assessed by the Children’s Depression Inventory 2, and depressive disorders, as assessed by the semistructured interview Kiddie Schedule for Affective Disorders and Schizophrenia (K-SADS) [9,10]. Despite this result, a significant number of adolescents were not free of depressive symptoms at posttreatment (24%), the effects were moderate, the dropout was high (57%), and it was hard to motivate patients to finish the treatment. Therefore, it is important to develop an attractive and more suitable treatment for adolescents who do not prefer to visit a mental health professional each week. Exploring possibilities to improve existing treatments in an innovative manner with recently developed technologies to reach out to patients may prove a fruitful strategy.

One way to improve the flexibility and attractiveness of treatment of depressed adolescents is to offer online CBT. Earlier research showed that online CBT and face-to-face CBT had, on average, similar effects in the treatment of depressive disorders in adults [11-14]. It is suggested that online interventions can increase treatment motivation and expectancies for some patients and might decrease resistance because they can be easily tailored to the adolescents’ needs and adjusted to their daily lives [15]. Another benefit is that online interventions seem more easily accessible for adolescents. Only 25% of the adolescents who suffer from depression receive treatment, so improving access to treatment is crucial [16]. Furthermore, adolescents appear to prefer to work autonomously on their treatment, which is typical for online interventions [15]. Online interventions increase independence and self-confidence of patients because patients can choose when and how often they work on the intervention. It also strengthens their competences [17] and can enhance social support by peers through online chat [18]. For these reasons, online CBT is a promising intervention.

However, when treating depression, face-to-face contact with therapists is strongly advised because the suicide risk has to be monitored. Therefore, treatment that is only offered online, such as online CBT, might not be sufficient for depressed adolescents. There are several therapist-supported options that can be combined with online treatment. Some treatment protocols offer online CBT combined with support or guidance by a therapist through encrypted email or online chat, called guided CBT. Several systematic reviews found that therapist guidance in guided or blended CBT increases the effectiveness of online interventions and is also associated with higher completion of the treatment [11,12,19]. The so-called blended treatment protocols could even be more appropriate or adequate for depressed adolescents. In blended treatments, the online protocol and face-to-face sessions are fully integrated into one treatment protocol. These blended treatment protocols might contain the optimal combination of flexibility and attractiveness for adolescents and possibilities to monitor progression and risk for therapists during the face-to-face sessions.

Health care costs of depression treatment could potentially be reduced by offering CBT in a blended form compared with face-to-face CBT or TAU because some face-to-face sessions with a therapist are replaced by an online program. Fewer face-to-face sessions per patient are needed; therefore, therapists can treat more patients, which in turn can decrease the duration of waiting lists [17]. Both improving access and reducing waiting lists might allow more patients to benefit from treatment and, eventually, societal costs may be reduced as well [20]. In sum, blended treatment, defined as a combination of online treatment and face-to-face sessions, might be the solution to optimize the effectiveness of CBT for depressive adolescents by increasing motivation and expectancy, decreasing resistance (and dropouts), and tailoring the treatment [15,17], which will likely lead to reduced costs [20]. However, the effectiveness has never been studied before in clinically depressed adolescents who are referred for treatment [21]. The treatment protocol D(o)epression Blended was specifically developed to treat depressive disorders in clinically referred adolescents [22].

This study will examine the effectiveness and cost-effectiveness of D(o)epression Blended compared with D(o)epression face-to-face and TAU in clinically depressed adolescents. The adolescents in the latter two conditions already participated in a previous study comparing CBT with TAU using an RCT design [9,10]. Blended CBT treatment is expected to be more effective than TAU. Additionally, we expect blended CBT to be equally effective as face-to-face CBT. We also will study the intervention effect of depression severity, quality of life, suicide risk, and comorbidity. The costs of the interventions in the three conditions will be compared to analyze the cost-effectiveness. In addition to examining the effectiveness and cost-effectiveness, we will examine who the intervention is effective for by testing several moderators (age, gender, ethnicity, family income, alcohol and drug use, life events, degree of conflicts, parental depression, and other psychopathology) and how the intervention works by studying several mediators (negative automatic thoughts, cognitive emotion regulation, attributional style). Furthermore, treatment characteristics will be studied; that is, characteristics of the therapists, treatment expectancy, previous treatments, treatment satisfaction, therapeutic alliance, cooperation with treatment, and dropout rates to see whether the feasibility of D(o)epression Blended differs from face-to-face CBT and TAU, and whether these factors are related to the implementation and execution of the treatment program.

## Methods

### Design

This study will use a pragmatic quasi-experimental controlled trial design with one condition to evaluate the effectiveness and cost-effectiveness of blended CBT. Data from a previously conducted RCT, in which face-to-face CBT was compared to TAU, will be used to compare the different treatments. Therefore, this can be seen as a third additional condition to the previously conducted RCT.

**Figure 1:**
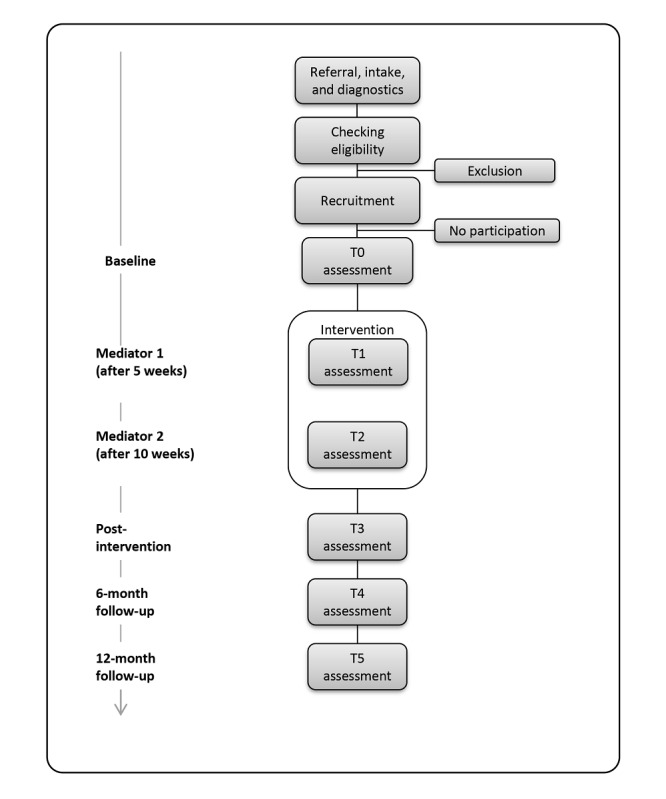
Overall study design. T0=baseline; T1=during the intervention after 5 weeks; T2=during the intervention after 10 weeks; T3=at 1 to 4 weeks postintervention; T4=at 6 months follow-up; T5=at 12 months follow-up.

A new cohort will be measured and treated in this study. The assessments and instruments will be identical to the previous study comparing CBT to TAU. Assessments will be conducted at baseline (T0), during the intervention after 5 weeks (T1), during the intervention after 10 weeks (T2), at 1 to 4 weeks postintervention (T3), at 6 months follow-up (T4), and at 12 months follow-up (T5). The overall study design is shown in Figure 1.

For the adolescents, it will take an estimated 60 minutes for T0; 40 minutes for T3, T4, and T5; and 30 minutes for T1 and T2. For parents, the measurements will take an estimated 45 minutes for T0, T3, T4, and T5, and an estimated 5 minutes for T1 and T2. For the therapist, T0 will take 3 minutes, and T1, T2, and T3 will take 1 minute to complete. Time estimations are based on earlier studies [10].

The study was approved by the medical ethics committee of the University Medical Centre Utrecht in the Netherlands (NL61804.041.17). Details are described according to the SPIRIT (Standard Protocol Items: Recommendations for Interventional Trials) guidelines [23]. The study was registered prospectively in the Dutch Trial Register (NTR6759) on October 16, 2017.

### Participants’ Eligibility

In total, 70 clinically depressed adolescents referred for treatment in one of nine participating mental health institutions will be asked to participate in this study, together with one of their parents. If the adolescent is 16 years or older, parents will only be approached with the adolescent’s permission. Inclusion criteria for the adolescents are (1) having a clinical diagnosis of a depressive disorder, (2) aged between 12 and 21 years, and (3) referred to one of the participating mental health institutions. Exclusion criteria are (1) acute risk of suicide, (2) drug abuse (as primary diagnosis), (3) pervasive developmental disorder (as primary diagnosis), (4) bipolar disorder (as primary diagnosis), (5) day care or admission to the clinical setting, and (6) insufficient knowledge of the Dutch language. If participants use medication, the dosage will be kept constant during the intervention.

### Recruitment

Adolescents with a depressive disorder referred for treatment in psychiatric care and their parents will be informed about the study and asked to participate. Researchers will obtain written informed consent from adolescents, and parents when adolescents are younger than 16 years, before enrolling participants in this study. Additional written informed consent will be obtained from parents when they participate in the study. All adolescents meeting the eligibility criteria and willing to participate will be assigned to the D(o)epression Blended intervention.

### Sample Size

The sample size (N=70) is equal to the sample size of the RCT that was already conducted in which D(o)epression face-to-face was compared with TAU. The data from this study will be compared with data from that RCT. The power analysis used in the previously conducted RCT was based on the difference between CBT and TAU at posttreatment. A meta-analysis on the effectiveness of CBT showed an effect size of 0.53 for CBT when delivered face-to-face [7]. Based on a meta-analysis on online interventions for depression (among which preventive interventions), the effect size was 0.76 [14]. Based on the literature, we assume that the effect size of blended CBT is equal to face-to-face CBT. We hypothesize that D(o)epression Blended is more effective than TAU. Furthermore, we will exploratively compare D(o)epression Blended to D(o)epression face-to-face. To detect the difference in depression diagnoses between the conditions of the previous study comparing blended CBT with TAU and this study, assuming alpha=.05 with a power of (1-β)=0.80 and dropout of 20% (power calculations in Stata), 70 adolescents will be included in this study.

### Intervention

D(o)epression Blended [22] is an adaptation of the Dutch D(o)epression face-to-face program [24]. D(o)epression face-to-face is an individual CBT program based on the evidence-based treatment program Coping with Depression course for Adolescents [24]. All 15 sessions of the original D(o)epression face-to-face protocol were adapted for use in an online environment in four modules. Major adaptations included a shortening of the wording, more use of attractive visuals, and the possibility to interact online with the therapist. The online program can be combined with a minimum of 5 and maximum of 15 face-to-face sessions. The face-to-face sessions last 45 minutes each, and the adolescent and the therapist are present. Face-to-face sessions are scheduled when the adolescent starts with a new online module and at the end of the treatment, as is advised in the manual. More face-to-face sessions can be added when the therapist considers this helpful or necessary, with a maximum of 15. Additionally, there is unlimited contact between the adolescent and therapist through a chat function within the program. During the face-to-face contacts, online modules are introduced, the therapist invests in the therapeutic relationship, exercises are practiced, and suicide risk is examined. There are also two face-to-face contacts with the parents (when parents are involved): after 3 weeks and after the start of the fourth module. Parents receive psychoeducation, information about CBT, and suggestions on how parents can contribute to the treatment. All CBT therapists who will offer the D(o)epression Blended as treatment were trained by a registered clinical psychologist in how to provide blended treatment and in working with this specific blended treatment protocol in a 1-day training session.

The content of D(o)epression Blended is the same as that of the face-to-face intervention and consists of the following components: psychoeducation, setting realistic goals, self-monitoring, activation, improvement of social and communication skills, relaxation skills, cognitive restructuring, role play, problem-solving skills, and relapse prevention [25]. These components are divided into four online modules, which are offered sequentially and are called Start, Do, Think, and Future. The monitoring of mood and activities is completely online. The exercises are introduced during the face-to-face contacts and are practiced online and at home, which increases the generalizability to real-life situations. The therapist receives automatically generated prompts when the adolescent has completed a task and can view the content of the task. The online environment offers convenience, autonomy, and flexibility because adolescents can tailor their treatment preferences, such as pace, frequency, and location. This means that blended treatment is vastly different from regular treatment and allows for tailoring of the treatment to the preferences of each individual.

The control condition is TAU, which consists of a broad range of different treatments. Mental health institutions offer interpersonal therapy, family therapy, parent counseling, medication, mindfulness training, ACT (acceptance commitment therapy), psychodynamic therapy (short duration), (nondirective) counseling, creative therapy, and running therapy. For research purposes, CBT is not allowed within TAU.

### Study Outcome Measures

For an elaborate overview of study variables, see Table 1.

**Table 1:** Overview of study variables.

Type of variable and concept		Instrument	Source			Assessment					
			Adolescent	Parent	Therapist	T0^a^	T1^b^	T2^c^	T3^d^	T4^e^	T5^f^
**Primary outcome**											
	Depression diagnosis	Kiddie Schedule for Affective Disorders and Schizophrenia	x	x		x			x	x	x
**Secondary outcomes**											
	Depression symptoms	Child Depression Inventory 2	x	x		x	x	x	x	x	x
	Depression severity	K-SADS	x	x		x			x	x	x
	Depression severity	Clinical Global Impression-Severity			x	x	x	x	x		
	Depression improvement	Clinical Global Impression-Improvement			x		x	x	x		
	Global functioning	Children Global Assessment Scale			x	x	x	x	x		
	Suicide risk taxation	Suicide Risk Taxation	x			x			x	x	x
	Comorbidity	Youth Self-Report scale/ Child Behavior Check List	x	x		x			x	x	x
**Cost-effectiveness**											
	Quality-adjusted life-years	EuroQol Questionnaire (EQ-5D-Y)	x	x		x	x	x	x	x	x
	Costs	Cost diary		x		x			x	x	x
**Moderators**											
	Gender, age, ethnicity, education, income	Demographic Characteristics	x	x		x					
	School or work	School Questionnaire	x			x			x	x	x
	Alcohol and drugs	Alcohol and Drugs	x			x			x	x	x
	Life events	Life Event Scale	x	x		x			x	x	x
	Degree of conflicts	Network of Relationships Inventory	x	x		x			x	x	x
	Parental depression	Beck Depression Inventory		x		x			x	x	x
	Parental psychopathology	Adult Self-Report		x		x			x	x	x
**Mediators**											
	Negative automatic thoughts	Cognitive Negative Cognitive Error Questionnaire	x			x	x	x	x		
	Cognitive emotion regulation	Cognitive Emotion Regulation Questionnaire	x			x	x	x	x		
	Attribution style	Children’s Attributional Style Questionnaire	x			x	x	x	x		
**Treatment characteristics**											
	Gender, age, experience, specialization of therapist	Therapy Procedure Checklist			x	x					
	Expectancy of treatment	Parent Expectancies for Therapy Scale	x	x		x					
	Previous treatments	Vragenlijst Eerdere Hulp en Interventies [History of Treatments]	x	x		x					
	Satisfaction treatment	Service Satisfaction Scale	x	x					x		
	Therapeutic alliance	Therapeutic Alliance Scale for Children	x		x		x	x	x		
	Cooperation with treatment	Cooperation With Treatment scale			x		x	x	x		
	Therapy questions	Therapy Questions			x		x	x	x		
	Treatment integrity	Observation			x				x		

^a^T0=baseline.

^b^T1=during the intervention after 5 weeks.

^c^T2=during the intervention after 10 weeks.

^d^T3=at 1 to 4 weeks postintervention.

^e^T4=at 6 months follow-up.

^f^T5=at 12 months follow-up.

### Primary Outcome Measure

Presence of the diagnosis of depression will be measured by the Present and Lifetime version of KSADS (K-SADS-PL) [26,27]. This widely used semistructured diagnostic interview assesses a wide range of diagnoses (present and lifetime), including their severity, taking into account the view of the adolescent and the parent, and will be conducted by a trained independent research assistant. Concurrent validity of the K-SADS-PL is supported; interrater agreement is high (93%-100%), and test-retest reliability is excellent for present and lifetime diagnoses of major depression [26].

#### Secondary Outcome Measures

Depressive symptoms will be measured with the Dutch version of the self-report measure Child Depression Inventory 2 [28,29], and with the Child Depression Inventory 2 parent version [28] rated by the parent. The severity of the depression will be assessed using the K-SADS-PL [26]. Severity of depression will also be assessed with the Clinical Global Impression-Severity scale [30], and improvement of depression will be assessed with the Clinical Global Impression-Improvement scale [30], both rated by the therapist.

Global functioning of the adolescent will be assessed with the therapist-rated Children Global Assessment Scale [31,32]. Suicide risk will be assessed with the K-SADS-PL [26], as well as with a self-report questionnaire Suicide Risk Taxation, which focuses on the frequency of suicidal thoughts, wishes, plans, and actions over the past 2 weeks. Comorbidity and psychopathology will be assessed with the K-SADS-PL [26], and additionally with the Youth Self-Report scale [33,34], rated by adolescents, and with the Child Behavior Check List for parents [33,34].

### Cost-Effectiveness Measures

Health-related quality of life will be measured with the Dutch version of the EuroQol Questionnaire (EQ-5D-Y) [35] and with the parent-rated EuroQol Questionnaire (EQ-5D proxy version) [35], which will be used to calculate quality-adjusted life-years.

The economic evaluation will be performed by registration of health care usage and costs in a cost questionnaire with a recall period of 3 months, which will be filled out by parents (which is identical to the one used in the previous RCT). Health care usage and registered costs that will be assessed are direct health care costs (eg, costs of psychologist, general practitioner, online treatment program, and medication), direct non-health care costs (eg, informal care), indirect costs (eg, monetary value of production losses caused by absence and reduced productivity and school), and out-of-pocket costs (eg, own contribution and transport costs). Costs will be calculated from a mental health care view and a societal view. The units of health care usage (eg, contact with the general practitioner, a session with a psychologist, costs of the online program) will be multiplied by the standard cost price obtained from the Dutch costing manual [36].

#### Moderators

Demographic information about the adolescents and parents will be assessed at baseline. Alcohol and drug use of the adolescents will be registered. Life events (such as bereavement, maltreatment, and suicide attempts), the date of occurrence, and their impact on the adolescent’s well-being will be registered with the Life Event Scale [37] by the adolescent and parent. The Network of Relationships Inventory will be filled in by both adolescents and parents to measure the degree of conflicts, such as quarrels, irritations, and antagonism, in the child-parent relationship [38]. Depressive symptoms in parents will be assessed with the Dutch version of the Beck Depression Inventory, second edition [39]. Parental psychopathology will be assessed with the Adult Self-Report [40].

#### Mediators

Negative cognitive errors will be measured with the Negative Cognitive Error Questionnaire [41], which consists of the subscales underestimation of the ability to cope, personalizing without mind reading, selective abstraction, overgeneralizing, and mind reading. Cognitive emotion regulation will be assessed with the Cognitive Emotion Regulation Questionnaire [42] consisting of several strategies as a response to experiencing stressful events, namely self-blame, other blame, rumination, catastrophizing, positive refocusing, planning, positive reappraisal, putting into perspective, and acceptance. Attributional style will be measured with the self-report Children’s Attributional Style Questionnaire [43], which contains three dimensions of attribution, namely internal-external, stable-unstable, and global-specific.

### Treatment Characteristics

At baseline, demographic information and information about education and experience of therapists will be assessed. Treatment expectancy will be measured with the Parent Expectancies for Therapy Scale [44], which will also be rated by adolescents in a revised version. Previous treatments for the adolescents’ depression, such as CBT, pharmacotherapy, self-help treatments, or alternative therapies, will also be reported by the adolescents and parents with the inventory of History of Treatments [45]. Adolescents’ and parents’ satisfaction with treatment will be assessed with the Service Satisfaction Scale [46]. Therapeutic alliance will be reported by the adolescent and therapist using the Therapeutic Alliance Scale for Children [47]. The degree of cooperation with treatment, as observed by the therapist, will be measured with the Cooperation With Treatment scale [48]. The content of treatment will be assessed with some questions on the procedure during the therapy, such as techniques used and amount of contact through email and chat. Treatment integrity will be checked by recording two randomly chosen sessions per patient that will be observed and rated by trained psychologists. Ratings will be based on the quality of the therapist (eg, empathy, motivating patient), content of treatment (eg, following protocol, attaining goals), and structure of the session (eg, setting agenda, following time schedule). Items will be rated on a scale from 0 to 3, in which 0=absent, 1=minimal, 2=largely, and 3=maximal. A mean score for treatment integrity will be calculated.

### Analyses

Intention-to-treat (imputed data) analyses and completer-only analyses will be conducted. Results will be reported in accordance with the CONSORT (Consolidated Standards of Reporting Trials) 2010 Statement [49].

#### Preliminary Analyses

Possible baseline differences in demographic and diagnostic characteristics between the three conditions—(1) D(o)epression Blended condition, (2) D(o)epression face-to-face condition, and (3) TAU condition (the latter two conditions from the RCT, which has already been conducted)—will be checked by means of ANOVA, MANOVA, and chi-square analyses. If these variables show differences between the conditions, they will be entered as covariates in all models and regression models to test the effectiveness of the intervention.

#### Analysis of Primary Outcome

The primary outcome measure (ie, presence of depression diagnosis) is dichotomous and will be tested with logistic regression analyses comparing the percentage of depressive disorders in the D(o)epression Blended condition in this study to the percentage of depressive disorders in the D(o)epression face-to-face condition and the TAU condition from the previous RCT.

#### Analysis of Secondary Outcomes

The intervention impact on the secondary outcomes (depression symptoms, depression severity, depression improvement, quality of life, suicide risk, comorbidity) will be evaluated using linear mixed modeling with the baseline level of the concerning variable as the covariate.

#### Cost-Effectiveness Analyses

A cost-effectiveness analysis will be conducted by comparing the costs and effects of the D(o)epression Blended condition in this study to the costs and effects and effects of D(o)epression face-to-face and TAU. The economic evaluation will be executed in accordance with the CHEERS (Consolidated Health Economic Evaluation Reporting Standards) Statement [50,51], which has been successfully executed previously in an economic evaluation of treatment of anxiety disorders in children [52]. The costs of blended CBT versus face-to-face CBT and TAU will be expressed in (1) incremental costs per adolescent with a depression in full remission (based on the K-SADS-PL) and (2) incremental costs per quality-adjusted life year (based on the EuroQol). To gain insight into the uncertainty surrounding subtotal and total costs, and due to highly skewed cost distributions, bootstrap simulations (1000 replications) will be conducted. Bootstrap simulations will be conducted to quantify the uncertainty around the incremental cost-effectiveness ratio [53], yielding information about the joint distribution of cost and effect differences. The bootstrapped cost-effectiveness ratios will be subsequently plotted in a cost-effectiveness plane. The bootstrapped incremental cost-effectiveness ratios will also be depicted in a cost-effectiveness acceptability curve showing the probability that a condition is cost-effective using a range of ceiling ratios. Also, sensitivity analyses will be performed to test the robustness of the results. The cost-effectiveness analyses will be done separately from a mental health perspective and a societal perspective over a period of 6 months and 1 year.

#### Analyses of Moderators

We will conduct a series of a priori planned moderator analyses to see if the intervention effect is moderated by the following moderators: adolescent characteristics (age, gender, ethnicity, family income), alcohol and drug use, life events, degree of conflicts, parental depression, and parental psychopathology.

#### Analyses of Mediator

Determining possible mediators that influence the effect of the intervention helps to identify the working mechanisms of the blended intervention. More specifically, we will test if the intervention effect on the presence of a depression diagnosis is mediated by changes in negative cognition, cognitive emotion regulation, and attributional style. The mediation analyses will be performed in Mplus [54], where indirect effects will be tested with bootstrap methods.

#### Other Analyses

We will also conduct analyses to see whether the feasibility of D(o)epression Blended differs from face-to-face CBT and TAU on treatment expectancy, previous treatments, satisfaction with treatment, cooperation with treatment, relationship with the therapist, treatment integrity, and dropout rate, and whether these factors are related to the implementation and execution of the treatment program.

## Results

### Overview

The primary outcome is the presence of a depression diagnosis at 12-month follow-up. Several secondary outcomes will be measured, such as depressive symptoms, quality of life, and suicide risk. Costs and effects in both conditions will be compared to analyze cost-effectiveness. Further, moderating (age, gender, alcohol and drug use, parental depression, and other psychopathology) and mediating (negative automatic thoughts, cognitive emotion regulation, attributional style) effects will be tested. Also, treatment characteristics will be studied, such as characteristics of the therapists, treatment expectancy, and therapeutic alliance. The dropout rate and treatment characteristics will be measured to study the feasibility of blended CBT.

### Ethics Approval and Consent to Participate

The study will be conducted according to the principles of the World Medical Assembly Declaration of Helsinki (2013) and in accordance with the Dutch Medical Research Involving Human Subjects Act (in Dutch: WMO) and other guidelines, regulations, and acts. Ethical approval has been obtained after extensive peer review by the medical ethics committee University Medical Centre Utrecht, The Netherlands (NL61804.041.17). The study is registered in the Dutch Trial Register (Trial ID: NTR6759). The study results will be reported in accordance with the CONSORT 2010 statement for reporting randomized trials [49]. Written informed consent to participate in the study will be obtained from adolescents and parents.

### Funding Status

This research was funded in December 2016 by the Dutch Organization for Health Research and Development ZonMW (grant number 70-72900-98-16144).

### Recruitment

The trial began recruiting participants in November 2017, and end of study enrollment was March 2019. At this time, 42 children have been recruited.

### Trial Status

We are in the process of conducting the 6- and 12-month follow-up assessments. The first results are expected to be submitted for publication in 2020.

### Datasets

The datasets used and/or analyzed during this study will be available from the corresponding author on reasonable request.

## Discussion

### Summary

This study protocol describes a pragmatic quasi-experimental controlled trial investigating the effectiveness and cost-effectiveness of a blended CBT treatment protocol in clinically depressed adolescents. Results of D(o)epression Blended will be compared with D(o)epression face-to-face and TAU. Data from the latter two conditions already have been collected in a previous study comparing CBT with TAU using an RCT design [9,10]. Testing the effectiveness of blended treatment is in accordance with the suggestions made by Calear and Christensen [55] and Richardson et al [56] to compare a blended treatment, such as blended CBT, to other active interventions, such as face-to-face CBT and TAU. Furthermore, we will examine the role of several moderators (comorbidity, depression severity, age, ethnicity, gender, family income, parental psychopathology) and mediators (negative automatic thoughts, cognitive emotion regulation, attributional style) to study who the intervention is effective for and how the intervention works. In this way, the study responds to unanswered questions about the moderators and mediators of blended interventions [6,57]. In addition, important factors in blended interventions, such as the dropout rate, feasibility, treatment expectancy, satisfaction with treatment, cooperation with treatment, relationship with the therapist, and treatment integrity will be studied.

### Strengths and Limitations

One of the strengths of this study is that it is carried out in mental health institutions and that the targeted population of this study consists of adolescents referred for treatment of their depressive disorder. Participants were not recruited for treatment for the purpose of this study. This means that conclusions from the results of this research can be generalized to real-life mental health care. Additionally, the study will be conducted in several mental health care centers in the Netherlands. Therefore, our findings will not be based on the results of only one mental health institution, which adds to the generalizability of the results. Another strength is that we will compare a blended CBT treatment protocol to active treatment conditions: face-to-face CBT and TAU. Moreover, assessments will be based on multiple informants (ie, adolescents, parents, and therapists).

This innovative blended treatment has considerable potential. It incorporates the positive characteristics of online interventions, such as high accessibility, and the potential to be easily tailored to the daily lives of adolescents. To our knowledge, this is the first study on the effectiveness of blended CBT treatment of depressive disorders in adolescents. Therefore, we can uniquely contribute to the crucial evidence-based improvement of treatment protocols in mental health care.

A limitation is that the study is not designed as an RCT. In this study, we will recruit participants for the blended CBT condition only. The results of these participants will be compared with participants recruited in the earlier conducted RCT, in which face-to-face CBT was compared to TAU. Since there is no randomization in the current trial, it cannot be ruled out that there are slight differences in the participants, therapists, or societal context. We will try to keep this potential threat to internal validity as low as possible by working with the same inclusion and exclusion criteria for participants and with the same or similar mental health institutions. However, this pragmatic quasi-experimental design will deliver preliminary evidence and very relevant information on the effectiveness and cost-effectiveness of blended CBT in routine care.

### Implications for Practice

If the blended CBT program D(o)epression Blended proves to be effective and cost-effective in treating depressive disorders in adolescents in mental health care, then D(o)epression Blended could be widely implemented in mental health institutions. This could expand the treatment choices of depressive disorders in adolescents and improve the potential to tailor and personalize the treatment.

## Appendix

Multimedia Appendix 1 Funder peer-review report (ZonMw).

## Abbreviations

CBT: cognitive behavioral therapy
K-SADS: Kiddie Schedule for Affective Disorders and Schizophrenia
K-SADS-PL: Kiddie Schedule for Affective Disorders and Schizophrenia-Present and Lifetime Version
RCT: randomized controlled trial
TAU: treatment as usual
