# Supplementary material for: Effectiveness and Cost-Effectiveness of Blended Cognitive Behavioral Therapy in Clinically Depressed Adolescents: Protocol for a Pragmatic Quasi-Experimental Controlled Trial
Source: JMIR Res Protoc. 2019 Oct 7;8(10):e13434. doi: 10.2196/13434 (PMC6803889; doi:10.2196/13434)
Supplement: Multimedia Appendix 1 [file resprot_v8i10e13434_app1.pdf]

ZonMw

ZonMw stimuleert
gezondheidsonderzoek en
zorginnovatie

Laan van Nieuw Oost-Indië 334
2593 CE Den Haag
Postbus 93245
2509 AE Den Haag
Telefoon 070 349 51 11
Fax 070 349 51 00
www.zonmw.nl
info@zonmw.nl

Dossiernummer
70-72900-98-16144
Ons kenmerk
2016/27624/ZONMW

Universiteit Utrecht
Faculteit Sociale Wetenschappen
Capaciteitsgroep Pedagogiek en Onderwijskunde
Mevrouw Drs. J.F.M. Neefjes
Postbus 80140
3508 TC UTRECHT

Datum
20 december 2016

Contactpersoon
Jojanneke Hillmann
Telefoon 070 349 52 67
Hillmann@zonmw.nl

Onderwerp
Honorering van uw subsidieaanvraag, projectnummer 729410007

Geachte mevrouw Neefjes,

Op 29 september 2016 heeft u bij ZonMw een subsidieaanvraag ingediend met de titel: *Doepressie blended; innovatief e-mental health programma voor klinisch depressieve adolescenten op maat!* binnen de subsidieronde 'programmalijn 4b: kennisontwikkeling over digitale innovaties die de kwaliteit van zorg in het jeugddomein verbeteren'.
Met genoegen laat ik u weten dat de werkgroep van het programma Effectief werken in de jeugdsector een positief oordeel heeft over uw aanvraag. Dat betekent dat ZonMw u de subsidie zal toekennen. In deze brief leest u hoe ZonMw tot dit oordeel is gekomen en wat u moet doen voordat uw project van start kan gaan.

Beoordeling
Uw aanvraag is beoordeeld op relevantie voor deze oproep van het programma en op kwaliteit. Voor deze ronde van het programma ontving ZonMw 20 subsidieaanvragen, waarvan er 9 wordt gehonoreerd. De beoordelingsprocedure was als volgt:

Relevantie van de aanvraag
De werkgroep van het programma Effectief werken in de jeugdsector heeft de relevantie van uw subsidieaanvraag voor deze oproep van het programma beoordeeld. Het eindoordeel over de relevantie van uw subsidieaanvraag voor het programma luidt: **relevant.**

Hierbij zijn de volgende opmerkingen van belang:
- De subsidieaanvraag voldoet aan de voorwaarden en doelstellingen van de subsidieoproep en de programmadoelstellingen.

Kwaliteit van de aanvraag
De werkgroep van het programma Effectief werken in de jeugdsector heeft ook een eindoordeel over de kwaliteit van uw subsidieaanvraag gegeven. Dit oordeel is gebaseerd op uw aanvraag, de beoordeling hiervan door referenten en uw reactie. Het eindoordeel over de kwaliteit van uw subsidieaanvraag luidt: **goed.**

De werkgroep geeft de volgende argumenten voor het eindoordeel:
- Met de aandachtspunten die de werkgroep u heeft meegegeven bij het positieve advies voor het uitwerken van het projectidee naar een subsidieaanvraag is over het algemeen goed rekening gehouden.

Onze hoofdopdrachtgevers zijn VWS en NWO

- De werkgroep had meer aandacht willen zien voor de kansen en belemmeringen van het onderzoek. De indruk bestaat dat er wat te luchtig gedacht wordt over de werving van 70 jongeren. Hoe dat aangepakt gaat worden is niet helemaal duidelijk. Het advies om een meer passende en hogere incentive te geven aan gezinnen is niet overgenomen. De werkgroep betwijfelt of de hoogte van de incentive in overeenstemming is met de tijdsinvestering van jongeren en ouders.
- De training aan therapeuten is duidelijk beschreven in de aanvraag. De werkgroep wil u meegeven om jongeren/ervaringsdeskundigen zelf te betrekken bij de training door hen samen met de gedragswetenschappers de training te laten verzorgen. Dan krijgen de therapeuten echt ervaring vanuit het perspectief van jongeren. Jongeren zelf kunnen het beste vertellen hoe je jongeren aanspreekt.

Op basis van beide eindoordelen heeft de werkgroep van het programma Effectief werken in de jeugdsector vervolgens een rangschikking van alle aanvragen die voor honorering in aanmerking kwamen gemaakt. Op grond hiervan heeft ZonMw uw aanvraag gehonoreerd.

We verzoeken u in het voortgangsverslag (halverwege de projectperiode) expliciet te rapporteren over:
- De hoogte van de incentive in relatie tot de tijdsinvestering van de deelnemende gezinnen.
- De werving van de 70 jongeren en hoe dit aangepakt wordt.
- Het mogelijk betrekken van jongeren/ervaringsdeskundigen bij de training aan therapeuten.
- Hoe de deelnemende jongeren en ouders blijvend betrokken en geïnformeerd worden over de voortgang.

Financiering

Hoogte subsidiebedrag
De financiële bijdrage van ZonMw voor uw project bedraagt maximaal € 199.875,- voor de duur van maximaal 30 maanden. Dit bedrag is inclusief eventueel verschuldigde BTW.

Subsidievoorwaarden
Zoals u weet zijn aan de financiering voorwaarden verbonden. Deze subsidievoorwaarden kunt u downloaden via de website van ZonMw:
www.zonmw.nl/subsidievoorwaarden
Ik wil u erop wijzen dat ZonMw pas een voorschot uitkeert als aan alle eisen voor het uitvoeren van het onderzoek is voldaan. Ik raad u dan ook aan eventuele procedures hiervoor tijdig te starten. Denkt u bijvoorbeeld aan een positief oordeel van een erkende medisch-ethische toetsingcommissie (METC), de Centrale Commissie Mensgebonden Onderzoek (CCMO), een projectvergunning van de Centrale Commissie Dierproeven (CCD), of een vergunning krachtens de Wet op het Bevolkingsonderzoek (WBO). Als u niet zeker weet of uw project dergelijke verklaringen of vergunningen nodig heeft, kunt u dit nagaan bij de betreffende instanties.

Integriteit
Artikel 2, lid 3 van de Subsidiebepalingen van ZonMw impliceert dat de nationaal en internationaal aanvaarde normen van wetenschappelijk handelen worden nageleefd zoals neergelegd in de Nederlandse Gedragscode Wetenschapsbeoefening (VSNU, laatste herziene versie 31 oktober 2014), dan wel vergelijkbare codes voor niet-universitaire instellingen. In geval van (mogelijke) schending van voornoemde normen bij een door ZonMw gefinancierd project, dient ZonMw hiervan onverwijld op de hoogte te worden gesteld en dienen alle ter zake relevante documenten aan ZonMw te worden overgelegd.

ZonMw bepaalt dat de bijlage Akkoord bekostiging wetenschappelijk onderzoek 2008 en het addendum, conform artikel 7 van het akkoord niet integraal van toepassing zijn op deze subsidie. Deze worden zoveel mogelijk analoog toegepast voor zover het akkoord of het addendum niet strijdig zijn met de Algemene subsidiebepalingen van ZonMw. De Algemene subsidiebepalingen van ZonMw zijn te allen tijde leidend. Zo zal ZonMw bijvoorbeeld altijd afrekenen op basis van werkelijke kosten.

Wat moet u doen?

Belangrijk: schriftelijke bevestiging binnen vier weken
ZonMw kan u een voorschot voor het eerste projectjaar verstrekken. Dit is echter pas mogelijk als u heeft ingestemd met de subsidievoorwaarden en het project daadwerkelijk gestart is. Wilt u daarom voor **18 januari 2017** schriftelijk onderstaande informatie doorgeven aan ZonMw? Hiervoor kunt u gebruikmaken van het bijgevoegde meldingsformulier:

- uw instemming met de voorwaarden die van toepassing zijn op de toekenning van de financiële bijdrage;
- de startdatum van uw project;
- de bank- en referentiegegevens voor de betalingen van de subsidie;
- Ten aanzien van de goedkeuring van de METC of CCD:
    - Als geen verklaring(en) is vereist, stuurt u een schriftelijke bevestiging hiervan.
    - Als de verklaring(en) noodzakelijk is voor de start van het project, stuurt u de verklaring voor de start van het project aan ZonMw.
    - In het geval de verklaring(en) pas later in het project vereist is, geeft u aan wanneer de verklaring(en) nodig is. Dit is maximaal één jaar na de start van het project. ZonMw keert dan een voorschot uit voor het eerste projectjaar. Verdere voorschotten kan ZonMw alleen betalen als een kopie van de verklaring(en) is ontvangen.

Ik wijs u erop dat het project **uiterlijk 1 juni 2017** moet beginnen. Gaat het project later van start, dan vervalt de honorering van uw aanvraag. Hiervan kan alleen in zeer bijzondere gevallen worden afgeweken.

Publiekssamenvatting
ZonMw publiceert alle gehonoreerde projecten op haar website met een leesbare Nederlandse samenvatting. Deze is bedoeld voor een breed geïnteresseerd publiek met verschillende achtergronden, op taalniveau eind VWO. Zie de schrijfwijzer op http://www.zonmw.nl/nl/over-zonmw/logo-huisstijl.
Wij verzoeken u deze Nederlandse publiekssamenvatting zo spoedig mogelijk, maar tenminste binnen vier weken (vóór 18 januari 2017) na dagtekening van deze brief aan te leveren. Hiervoor kunt u in ProjectNet het tekstvak Publiekssamenvatting gebruiken (maximaal 1000 karakters, inclusief spaties).

Voortgangsverslag
ZonMw wil graag op de hoogte blijven van de voortgang van uw project. ZonMw werkt met een verkorte voortgangsrapportage die u halverwege het project indient (tenzij anders bepaald). Van het programmasecretariaat ontvangt u te zijner tijd het verzoek om een voortgangsrapportage in te dienen.
Daarnaast bent u verplicht tussentijdse wijzigingen te melden aan ZonMw. Pas na goedkeuring door ZonMw zijn de wijzigingen toegestaan.

Kennisbenutting
Resultaten van het project kunnen toepassing vinden in de praktijk, maar ook een rol spelen bij het maken van beleid, een volgende stap vormen in een wetenschappelijke carrière of de basis vormen voor een nieuw project. Om aan te geven wat er met de resultaten gebeurt, stellen wij u in voortgangs- en eindverslag

diverse vragen over verspreiding- en implementatie. Ook dienen publicaties over en resultaten van het project tot vier jaar na afronding via ProjectNet aan ZonMw te worden aangeboden. Daarnaast bent u verplicht om ZonMw in deze periode te informeren over het gebruik van de resultaten.

Mocht u nog vragen hebben over deze brief, neemt u dan gerust contact op met de medewerker die in het briefhoofd vermeld staat. Als u ontevreden bent over de wijze waarop ZonMw uw aanvraag heeft behandeld, kunt u een klacht indienen (zie hieronder). Vermeld in uw communicatie met ZonMw altijd het projectnummer. Nu uw aanvraag gehonoreerd is, vervalt het oorspronkelijke nummer en geldt het nieuwe projectnummer: **729410007**.

Ik wil u nogmaals feliciteren met de honorering van uw subsidieaanvraag. Veel succes bij de uitvoering van uw project!

Met vriendelijke groet,
namens het bestuur,

i.a.

Henk J. Smid
directeur

Bijlage(n)
Meldingsformulier start project (bestemd voor hoofdaanvrager/projectleider penvoerder)

Kopie
Radboud Universiteit Nijmegen, mevrouw dr. D.H.M. Bodden

Tegen deze beschikking kunt u bezwaar maken. In dat geval stuurt u binnen zes weken na de dag waarop het besluit bekend is gemaakt een bezwaarschrift aan het bestuur van ZonMw, t.a.v. Commissie Bezwaarschriften ZonMw, Postbus 93 245, 2509 AE Den Haag. Meer informatie over signaleren, klagen en bezwaar maken? Raadpleeg de website: www.zonmw.nl/signalerenklagenbezwaarmaken

MELDINGSFORMULIER START PROJECT ZonMw

Doepressie blended; innovatief e-mental health programma voor klinisch depressieve adolescenten op maat!

Dit formulier inzenden binnen vier weken na de dagtekening van honoreringsbrief. Graag met blauwe of zwarte pen invullen in blokletters.

1.	Projectnummer[a]	729410007 (70-72900-98-16144)
2.	Naam Projectleider	Dr. D.H.M. Bodden
3.	Toegekende subsidie	€ 199.875,-
	**START PROJECT**	
4.	Startdatum	
5.	Looptijd (in maanden)	30 maanden
6.	Einddatum	
	**BANKGEGEVENS**	
7.	IBAN	
	BIC	
8.	Ten name van:	
	te:	
9.	Bij betaling te vermelden kenmerk:	
	**CORRESPONDENTIEADRES T.B.V. FINANCIELE ZAKEN**	
10.	Naam Instelling/Afdeling	
11.	E-mailadres t.b.v. ZonMw betaalspecificaties	
12.	Adres/Postbus	
13.	Postcode/Plaats	
	**METC/CCMO/CCD/WBO**	
14.	☐ Er is geen positief oordeel van METC/CCMO[b], of een (project)vergunning van CCD/WBO[b] vereist.	
15.	☐ Er is een positief oordeel METC/CCMO[b], of een (project)vergunning van CCD/WBO[b] vereist. Ik stuur een kopie van het document mee bij dit formulier.	
16.	☐ Er is in de looptijd van het project een positief oordeel van METC/CCMO[b] of een (project)vergunning CCD/WBO[b] nodig. Maximaal één jaar na de start van het project stuur ik een kopie van het document op.	
	**DATAMANAGEMENT**	
17.	☐ Ik ga mijn datamanagementplan opstellen in samenwerking met:	
18.	☐ Ik ga geen nieuwe data verzamelen.	

Door ondertekening verklaart zowel de projectleider als de instelling zich akkoord met de ZonMw subsidievoorwaarden en de in toekenningsbrief opgenomen specifieke voorwaarden/uitsluitingen.

Handtekening projectleider:

Naam:
Dr. D.H.M. Bodden

Datum:

Handtekening namens bestuurlijk verantwoordelijke:

Naam: Drs. J.F.M. Neefjes

Functie:

Datum:

---

[a] Bij financiering uit additioneel VIMP-geld, hier ook het oorspronkelijke projectnummer vermelden.

[b] Doorhalen wat niet van toepassing is.

Referent B.2016.01AB7

1. Uitwerking vraagstelling 5 en 6
De hoofdvraagstelling is het bepalen van de (kosten)effectiviteit. De rol van non-specifieke therapie variabelen (vraagstelling 5) en de individuele verandertrajecten (vraagstelling 6) worden exploratief onderzocht. De non-specifieke therapie variabelen gaan we vergelijken voor de 3 groepen (blended, face-to-face en CAU) m.b.v. ANCOVA's om vervolgens na te gaan met regressie analyses of deze non-specifieke variabelen de behandeleffecten verschillend voorspellen. Normaliter wordt er in effect onderzoek gekeken of de groep als geheel gemiddeld genomen effecten laat zien. M.b.v. Latent Class Growth Analyses (zie Nagin & Odges, 2010) wordt er gekeken of er bepaalde groepen te onderscheiden zijn waarbij Doepressie-Blended beter werkt en op welk moment in de behandeling Doepressie-Blended het meest effectief is (begin, midden of eind van de behandeling).

1.2 Verschil in behandelvormen en kosten digitale innovatie
Doepressie-blended is gebaseerd op CGT die deels online gevolgd worden en gedeeltelijk face-to-face. Doepressie-face-to-face is gebaseerd op CGT waarbij alle sessies face-to-face gedaan worden. CAU is de reguliere zorg voor depressieve jongeren en omhelst alles behalve CGT (zoals IPT, gezinstherapie, ouderbegeleiding, medicatie, etc.).
Doepressie-blended is onderdeel van een geïmplementeerd behandelplatform met verschillende interventies ontwikkelt door Jouw Omgeving (JO) op verzoek van en gefinancierd door de K7 psychiatrische kinder en jeugd instellingen (K7). JO zorgt voor het onderhouden, door ontwikkelen en borgen van de interventie. Gebruikskosten worden verrekend en meegenomen in dit onderzoek.

1.3 Onduidelijkheid Metingen
Er is een voormeting, nameting, 6-maanden follow-up en 12-maanden follow-up. Daarnaast zijn er 2 mediator-metingen tijdens de behandeling. Alle vragenlijsten zijn in de eerdere genoemde RCT reeds gebruikt en goedgekeurd door de METC. Een overzicht met daarin alle vragenlijsten, informanten en meetmomenten is beschikbaar maar mocht niet toegevoegd worden (communicatie met Jojanneke Hillman).

-Rol adviesraad
Bij start, bij de uitvoer en aan het einde van het onderzoek zal de adviesraad bij elkaar komen en gevraagd worden om (1) verbeteringen aan te geven bij het implementeren van het onderzoek, (2) aanbevelingen mbt de uitvoer van het onderzoek, (3) resultaten interpreteren en (4) aanpassingen van Doepressie-blended en (5) nieuw onderzoek.

-Inclusie aantal en aantal instellingen
Er nemen 7 instellingen deel aan deze studie; Accare, De Bascule, Curium, GGZ Oost Brabant, Herlaarhof, Praktijk Appelboom en Triversum, waarvan 4 behoren tot de K7. Per instelling zullen 6 tot 14 jongeren geïncludeerd worden. In totaal worden 70 jongeren geïncludeerd.

1.4 Rollen en functies

Per instelling zullen een onderzoeksassistent, een instellingscoördinator en behandelaren meedoen aan het onderzoek. De onderzoeksassistent voert de metingen en de KSADS interviews uit, de behandelaren voeren de blended behandeling uit. Gebaseerd op de geografische ligging van de instellingen, hebben we de onderzoeksassistenten verdeeld. Bij 4 instellingen, is er een gedeelde onderzoeksassistent die 2 instellingen ondersteunt. De instellingscoördinator zorgt voor de uitvoer van de studie binnen de instelling en begeleiding van onderzoeksassistent en behandelaren. De postdoc (omschreven in begroting als postdoc 3) is aangesteld bij de Universiteit Utrecht en zorgt voor de coördinatie, analyses en verslag legging. Hij zal regelmatig contact onderhouden met de instellingscoördinator en de onderzoeksassistent. Twee senior onderzoekers (omschreven als postdoc 1 en 2 in de begroting) helpen met coördinatie taken, begeleiden de postdoc en superviseren de therapeuten.

1.5 Planning onderzoek

- 1-6-2017: Training Doepressie-blended (voornamelijk therapeuten Herlaarhof en GGZ Oost Brabant)
- 1-7-2017: Training onderzoeksassistenten, bijeenkomst adviesraad en voorbereidingen implementatie
- 1-8 t/m 1-11 2017: Inclusie en Voormeting
- Vanaf 1-11-2017: Nametingen
- Vanaf 1-5-2018: 6-maanden follow-up metingen
- 1-7-2018: Laatste inclusie en voormetingen
- Vanaf 1-11-2018: 12-maanden follow-up metingen
- Van 1-7-2019 t/m 1-12-2019: Analyses, bijeenkomst adviesraad, publicaties en implementatie resultaten
- 1-11-2019: laatste 12-maanden follow-up metingen

3. *Begroting eigen bijdrage*
De eigen bijdrage bestaat uit €200.000 die de K7 geïnvesteerd hebben in de ontwikkeling.

Referent B.2016.01AB8

1,2 Betrokkenheid ouders
Ouders krijgen psychoeducatie over depressie en CGT (sessie 1) en ze worden betrokken bij de voortgang en evaluatie van de behandeling (sessie 12). Bovendien willen we de conditie gelijk houden aan Doepressie-face-to-face. Ook blijkt individuele CGT met aanvullende ouder sessies niet effectiever dan individuele CGT (Brent et al., 1997; Clarke et al., 1999).

1.3 Bias door geen randomisatie
Er wordt inderdaad niet gerandomiseerd maar iedere adolescent die voldoet aan de inclusie criteria krijgt de interventie aangeboden. Dus hier is geen selectiebias. De jongere kan inderdaad weigeren om de geïndiceerde interventie te volgen, de reden hiervan zal nagevraagd worden om te checken of de jongere een voorkeur heeft voor face-to-face behandeling. Deze procedure komt overeen met de procedure die we hanteerden in de eerder uitgevoerde RCT. Helaas hebben we destijds niet gevraagd naar affiniteit met technologie/e-health. Er kan dus

een cohort effect zijn, we zullen daarom de procedures zo veel mogelijk gelijk houden en baseline verschillen checken.

-Dappere kat besmetting
De verwachting is dat er weinig “besmetting” zal plaatsvinden omdat beide programma’s verschillen m.b.t. problematiek, leeftijdsgroep, opbouw en technieken. Dit wordt in de treatment integrity check meegenomen.

-Observaties en onafhankelijkheid
Observaties worden inderdaad gedaan bij de behandelsessies om de treatment integrity te meten. Verbetering van depressie wordt door verschillende informanten gerapporteerd namelijk kind (CDI en KSADS), ouder (CDI en KSDAS) maar ook de therapeut (CGI-I) en de onafhankelijke onderzoeksassistent (KSADS). Zo wordt onafhankelijkheid gewaarborgd.

-Exclusie IQ<70
Jongeren met IQ<80 worden geëxcludeerd vanwege de vorm van therapie en het invullen van de vragenlijsten.

- Kansen/belemmeringen implementatie
De problemen en feasibility van Doepressie-blended worden geïnventariseerd, gerapporteerd, bediscussieerd met de adviesraad en gebruikt voor verdere implementatie.

1.4 communicatielijnen
Zie Referent B.2016.01AB7 1.4 Rollen en functies

1.5 Geografische spreiding en opleiding behandelaars
De geografische spreiding is inderdaad een uitdaging maar door onderzoeksassistenten op de diverse locaties te vestigen (zoals in de eerdere RCT) worden problemen geminimaliseerd. Het onderhouden van contact en de dataverzameling vindt veelal online plaats. De interventie zal aangeboden worden door GZ psychologen maar ook door CGT therapeuten, overeenkomstig met de vorige RCT.

3.1 Rollen en functies
Zie Referent B.2016.01AB7, 1.4 Rollen en functies
De benchfee wordt gebruikt voor noodzakelijke bijscholing (zoals statistiek) om het onderzoek goed uit te voeren en voor congressen waar de resultaten van deze studie gepresenteerd worden.

Kortom, de inhoudelijke reactie van de referenten is positief en we hopen dat we hun additionele vragen afdoende hebben toegelicht.
